# Editorial: Purinergic Signaling and Inflammation

**DOI:** 10.3389/fimmu.2021.699069

**Published:** 2021-05-19

**Authors:** Xiaoyi Yuan, Davide Ferrari, Tingting Mills, Yanyu Wang, Agnieszka Czopik, Marie-Francoise Doursout, Scott E. Evans, Marco Idzko, Holger K. Eltzschig

**Affiliations:** ^1^Department of Anesthesiology, McGovern Medical School at UTHealth, Houston, TX, United States; ^2^Section of Microbiology and Applied Pathology, Department of Life Science and Biotechnology, University of Ferrara, Ferrara, Italy; ^3^Department of Biochemistry and Molecular Biology, McGovern Medical School at UTHealth, Houston, TX, United States; ^4^Department of Pulmonary Medicine, Division of Internal Medicine, The University of Texas MD Anderson Cancer Center, Houston, TX, United States; ^5^Department of Pulmonology, Medical University of Vienna, Vienna, Austria

**Keywords:** purinergic signaling, inflammation, mucosal inflammation, adenosine receptors, ATP receptors

Purine nucleotides and nucleosides are essential building blocks for cellular energy. Extracellular nucleotides and nucleosides signaling is increasingly recognized to control many other human physiological processes, including the pathogenesis of inflammatory diseases (**Figure 1**, material from Idzko et al.) (1). Adequate inflammatory responses are critical to fighting against invading pathogens and recovering from tissue injury. However, unresolved or chronic inflammation can cause tissue injury and disease pathogenesis (2, 3). The proper control of tissue inflammation requires synergistic action of many different pathways, with purinergic signaling playing diverse and important roles in this process (4–6). In this special Research Topic, the interaction between purinergic signaling and inflammation was highlighted by several original, review, opinion, and perspective articles.

**Figure 1:**
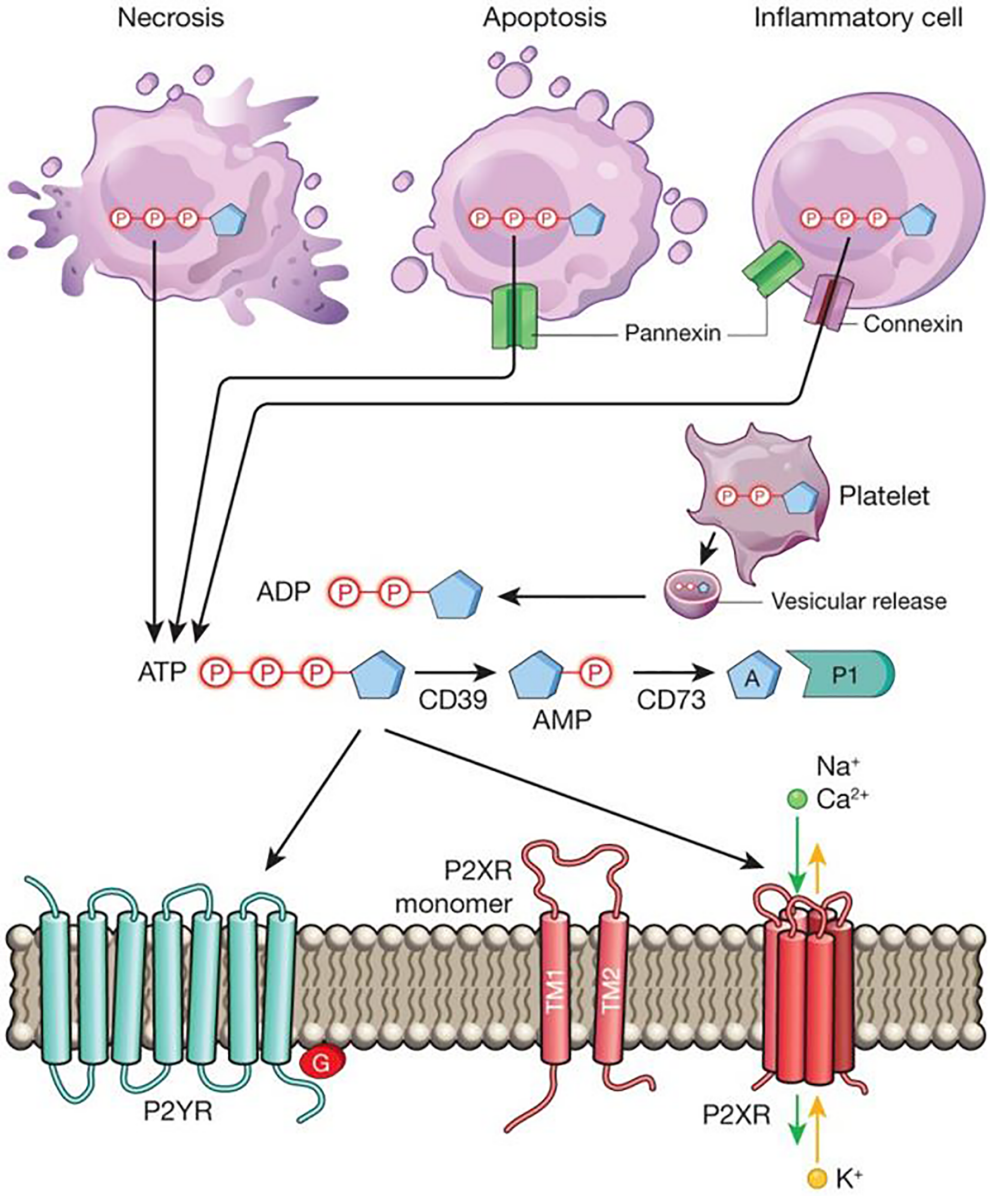
Extracellular nucleotide release and signalling during inflammation. During inflammation, multiple cell types release nucleotides, for example ATP or ADP, from their intracellular compartments into the extracellular space. Nucleotides can be released during mechanical injury, necrosis, apoptosis or inflammatory cell activation. Several molecular pathways have been implicated in this process, such as vesicular ADP release from platelets, pannexin-mediated ATP release during apoptosis, and connexin- or pannexin-mediated ATP release from inflammatory cells, such as neutrophils. Extracellular nucleotides function as signalling molecules through the activation of purinergic P2 receptors. These receptors can be grouped into metabotropic P2Y receptors (P2YRs; GPCRs with seven transmembrane-spanning motifs) or ionotropic P2X receptors (P2XRs), which are nucleotide-gated ion channels. Each P2XR is formed by three subunits (P2XR monomers), each of which consists of two transmembrane regions, TM1 and TM2. Binding of three molecules of ATP to the assembled P2X channel causes opening of a central pore. These conformational changes allow for flux of ions such as sodium (Na+), calcium (Ca2+) and potassium (K+) across the membrane. ATP signalling is terminated by the enzymatic conversion of ATP to adenosine through the ectonucleoside triphosphate diphosphohydrolase CD39 (conversion of ATP/ADP to AMP) and the ecto-5′-nucleotidase CD73 (conversion of AMP to adenosine). Similar to ATP, adenosine (A) functions as an extracellular signalling molecule through the activation of purinergic P1 adenosine receptors. Material from: Idzko et al. (1). Reprinted with permission.

Purinergic signaling orchestrates mucosal inflammation. An opinion article from Antonioli et al. encapsulates the contribution of adenosine system in many aspects of inflammatory bowel diseases (IBD), including intestinal inflammation, abdominal pain, and enteric dysmotility. Preclinical studies indicate that targeting A2A and A3 adenosine receptor has great therapeutic potential for IBD. While activation of those two adenosine receptors by selective agonists is beneficial in attenuating many aspects of IBD, this article highlights the need to develop novel, selective ligands on adenosine receptors. Relatedly, a mini-review article by Vuerich et al. describes how purinergic signaling controls gut inflammation. Indeed, many studies have indicated that therapeutic targeting of ATP (P1) receptor, adenosine (P2) receptor, and ENTPD1/CD39, and/or ecto-5′-nucleotidase (CD73) can directly modulate intestinal inflammation. The mini-review provides a concise overview of the current knowledge of purinergic-based therapy for IBD. Besides, an original article by Libera et al. elaborates on the role of CD39 and CD73 in IBD. The study compares the expression of CD39 and CD73 in T cell populations that include CD4, CD8, and γδ T cells in peripheral blood, as well as mucosal tissue from healthy individuals and IBD patients. Peripheral T cells have a CD39^low^CD73^high^ phenotype with high levels of IL-17A and IFNγ, while gut mucosal T cells have a CD39^high^CD73^low^ phenotype and low expression of IL-17A, IFNγ, and IL-10. These results suggest that CD39 and CD73 might be important for the phenotypic adaptation of T cells in the gut mucosal environment. Extending this to another organ system, the review article by Li et al. highlights the important role of adenosine signaling in the crosstalk between hypoxia and inflammation in lung injury. Hypoxia and inflammation are tightly linked together (7). Hypoxia signaling results in the activation of adenosine signaling *via* induction of A2A adenosine receptor and A2B adenosine receptors (8). Among these, the HIF/adenosine axis provides lung protection in acute respiratory distress syndrome though it also promotes inflammation and injury in chronic lung disease (9). Moreover, this article discusses the strategies to therapeutically target the adenosine signaling pathway in lung disease.

Inflammation is commonly observed in cancer and purinergic signaling is crucial in many aspects of cancer development. The review article by Steingold and Hatfield highlights the evidence to support targeting hypoxia-A2A adenosine receptor pathway to release the immunosuppression of anti-tumor T cells. The activation of hypoxia-adenosine-A2A axis in cancer leads to immunosuppression by inhibiting the effector function of T cells. Preclinical and clinical studies suggest that inhibitors targeting A2A, CD39/CD73, or hypoxia signaling can control cancer development when combined with immune checkpoint inhibitors. Moreover, a mini-review by Hamarsheh and Zeiser discusses the contradictory roles of NLRP3 inflammasome in cancer. NLRP3 inflammasome is canonically activated by danger-associated molecular patterns when concurrently exposed to a secondary signal, such as hypoxia, reactive oxygen species, or P2X7R activation. The article concludes that NLRP3 inflammasome acts as a double-edged sword in cancer and future studies should dissect the functional determining factors.

Purinergic signaling is important in modulating immune cell functions during inflammation. The review article by Ferrari et al. highlights how purinergic signaling shapes eosinophil phenotype to elicit pro-inflammatory or anti-inflammatory responses during homeostasis or pathological conditions. The article summarizes the diverse functions of eosinophils in the fight against invading microorganisms and allergic responses. Specifically, the article elegantly highlights how P2 receptors and P1 receptors differentially regulate eosinophil migration and function to exert pro-inflammatory or anti-inflammatory responses during tissue inflammation. The article concludes that P2 receptor inhibitors are potential therapeutic candidates in eosinophilic diseases, while further understanding about nucleotide stimulation of eosinophils in other inflammatory conditions such as cancer is needed. P2 receptor signaling is also crucial to the regulation of T cell function. A mini-review article by Ledderose and Junger describes the convergence of metabolic and purinergic signaling in the modulation of T cell function in host immune defense and inflammatory disorders. ATP accumulation is commonly observed during tissue injury and ATP can bind to P2X and P2Y receptors to modulate T cell function. This review provides a concise overview of how different P2 receptors interact with mitochondria to govern multiple aspects of T cell functions including T cell quiescence, migration, and formation of the immune synapse. Finally, the perspective article from Grassi further highlights the functions of P2X7 receptor in T cell regulation. The article concludes that P2X7 guides the development of γδ T cell in the thymus and promotes the development of Th1 and Th17 responses, conversion of Treg to Th17 cells, and cell death of Tfh cells in peripheral lymphoid organs. Demonstrating a particular role in intestinal immune homeostasis, microbiota-derived extracellular ATP results in P2X7 activation to induce cell death of effector T cells and, in turn, attenuates murine model of colitis.

The articles included in this Research Topic provide an inspiring overview of the interplay between purinergic signaling and inflammation. The collection of articles in the Research Topic also shed light on the importance and complexity of purinergic signaling in different disease settings, such as mucosal inflammation and cancer. Taken together, the papers presented in this Research Topic indicate that new therapeutic developments targeting purinergic signaling are needed to harness this pathway for the treatment of tissue inflammation.

## Author Contributions

XY drafted the manuscript. DF, MI, and HKE edited the topic and revised the manuscript. TM, YW, AC, M-FD, and SEE edited the manuscript. All authors contributed to the article and approved the submitted version.

## Funding

This work was supported by the American Thoracic Society Unrestricted Grant, American Heart Association Career Development Award (19CDA34660279), American Lung Association Catalyst Award (CA-622265), the Center for Clinical and Translational Sciences, McGovern Medical School Pilot Award (1UL1TR003167–01), and Parker B. Francis Fellowship to XY; National Institute of Health Grants R01HL154720, R01DK122796, R01DK109574, R01HL133900 and Department of Defense Grant W81XWH2110032 to HKE.

## Conflict of Interest

The authors declare that the research was conducted in the absence of any commercial or financial relationships that could be construed as a potential conflict of interest.
